# Comparison of peripheral iridectomy methods for posterior chamber phakic intraocular lens implantation in patients with brown irides

**DOI:** 10.1186/s12886-016-0229-x

**Published:** 2016-05-05

**Authors:** Yan Wu, Fei Han, Yan Quan, Wei Jiang, Hengdi Zhang, Tao Luo

**Affiliations:** Department of Ophthalmology, ChengDu Military General Hospital, ChengDu, 610083 Sichuan Province China; Department of Health, ChengDu Military General Hospital, ChengDu, 610083 Sichuan Province China

**Keywords:** Posterior chamber phakic intraocular lens, Peripheral iridectomy, High myopia, Dark-brown iris

## Abstract

**Background:**

We explore and compare the advantages and disadvantages of different operating methods for a peripheral iridectomy (PI) for phakic posterior chamber implantable contact lens (ICL) implantation in patients with dark-brown irides.

**Methods:**

Forty-six patients completed this prospective comparative study. Neodymium: yttrium-aluminum-garnet (Nd:YAG) PI was performed in 15 patients (30 eyes) 2 weeks prior to surgery (YAG PI group). Surgical PI was performed in 17 patients (34 eyes) 2 weeks prior to the ICL implantation (preoperative PI group), and intraoperative PI was performed during ICL implantation in 14 patients (28 eyes) (intraoperative PI group). The postoperative recovery of visual acuity, intraoperative complications, operation duration, and patients’ visual disturbances were compared.

**Results:**

Compared with the preoperative BCVA, the uncorrected visual acuity (UCVA) at 1 week was markedly restored in the preoperative PI group (*P* = 0.004). UCVA in the three groups of patients had all recovered well at 1 and 3 months after ICL implantation and were significantly better than the preoperative BCVA (all *P* < 0.01). In the YAG PI group, iris bleeding occurred in nine eyes (30.0 %) and 14 eyes (46.7 %) had pigment dispersion; these values were significantly higher than those in the preoperative PI group (5.9 and 14.7 %, respectively, both *P* = 0.01). In the intraoperative PI group, elevated high intraocular pressure occurred in four eyes (14.3 %), and eight eyes (28.6 %) had varying degrees of pigment dispersion after ICL implantation.

**Conclusions:**

For patients with dark-brown irides, surgical PI performed 2 weeks prior to the implantation facilitated better postoperative recovery of visual acuity.

**Trial registration:**

Current Controlled Trials ISRCTN35178162. Retrospectively registered March 4th, 2013.

## Background

Implantable contact lenses (ICLs) are phakic posterior chamber intraocular lenses used for the treatment of high myopia, hyperopia, and astigmatism, and are one of the major recent innovations in refractive surgery. Implantation of these lenses can significantly improve visual acuity and quality, [1] and their safety and efficacy have been confirmed.

In early studies of ICL surgery, there were reports [1–3] that peripheral iridectomy (PI) could effectively prevent pupillary block and subsequent high intraocular pressure (IOP) after ICL implantation. Currently, most surgeons perform preoperative neodymium:yttrium-aluminum-garnet (Nd:YAG) laser peripheral iridectomy (YAG PI), which is also recommended by the manufacturers of ICLs. However, the incidence of pupillary block remains at 4.0–11.1 % [4–6]. The timely application of YAG lasers, which enlarge the iris incision, or increasing the number of iris incisions can effectively relieve pupillary block and reduce IOP [4, 7]. Some studies suggested that PI effectively prevent pupillary block [8–10]. The size of the iris incision should be large but should not cause the iris to be penetrable by light or cause diplopia and other complications [8–10].

Most Asians have thick, dark-brown irides, which makes it difficult to create two unblocked and large holes with a YAG laser. Chun et al. [11] reported that in ten eyes of eight patients with dark-brown irides (12.3 %), narrowing or obstruction of the laser iridotomy of 50 % or more was observed 6 months postoperatively and required additional laser treatments. The reason for the narrowing or obstruction was dark-brown pigment epithelium regeneration; therefore, intraoperative PI was recommended. Pesando et al. [12] applied traditional intraoperative PI for patients with brown irides and achieved good results. In the present study, we performed a prospective comparison of different PI approaches for ICL implantation in patients with dark-brown irides. Our results provide a better understanding of the advantages and disadvantages of the various approaches.

## Methods

This study was a prospective comparative study retrospectively registered with Current Controlled Trials (ISRCTN35178162) on March 4th, 2013 (http://www.isrctn.com/ISRCTN351781620. The study was approved by the ethical committee of the ChengDu Military General Hospital (Sichuan, China). A written informed consent was obtained from participant. The study was carried out in accordance to the principle of the Declaration of Helsinki.

### Patient selection and grouping

From January 2011 to June 2012, 60 patients (120 eyes) with high myopia were treated at the ChengDu Military General Hospital and were enrolled in this study. Inclusion criteria were: 1) dark brown irides; 2) aged between 18 and 45 years; 3) high myopia, with preoperative diopter had to be between−9.50 D and−22.0 D (spherical equivalent); 4) inoperable by LASIK; 5) anterior chamber depth >2.8 mm; 6) no abnormalities of the cornea, angle structure, IOP, or peripheral retina on routine examination; 7) anterior chamber angle was open; and 8) endothelial cell count was >2500 cells/mm^2^. Patients were excluded if they had any other eye disorders (e.g., glaucoma, cataract, retinal degeneration, dry eye syndrome, etc.) or any systemic disorders that could affect the surgical outcomes (e.g., Sjogren’s syndrome, Behcet’s syndrome, etc.).

Finally, 58 patients (116 eyes) were included, and divided into three groups: 1) YAG PI before ICL implantation (Nd:YAG PI was performed 2 weeks prior to ICL implantation) group included 19 patients (38 eyes); 2) PI before ICL implantation (PI was performed 2 weeks prior to the ICL implantation) group included 20 patients (40 eyes); and 3) intraoperative PI during ICL implantation (PI was performed during ICL implantation) group included 19 patients (38 eyes).

### Surgery

Best-corrected visual acuity (BCVA) was assessed pre-operation. All YAG PI procedures and all PI and ICL implantations were performed by the same surgeon (Yan Wu). The ICMV4 ICL/T-ICL (Toric ICL) lens from STAAR Surgical Co. (Monrovia, CA, USA) were used in all patients. The operation duration was recorded.

YAG PI was performed 2 weeks prior to ICL implantation surgery. Pilocarpine drops (2 %) were applied three times for miosis 20 min prior to laser surgery. The two iridectomy holes were made at the 10:30 and 1:30 positions, separated by approximately 90°, in the middle of the peripheral areas under the eyelids. To prevent iris bleeding and pigment dispersion, standard argon laser settings (0.1–0.2 s duration, 50 mm spot size, 700 to 1500 mW) were initially used to form a crater in the iris stroma. After deep stromal iris penetration or the first plume of iris pigment epithelium release, the Nd:YAG laser (1.0 mJ, single burst, with power increased as needed) was immediately used to achieve iris perforation and remove the iris pigment epithelium. The diameter of each hole was approximately 0.8 mm. Complete penetration of the pigment epithelium was confirmed by transillumination.

For surgical PI, PI was performed 2 weeks prior to ICL implantation surgery. Pilocarpine drops (2 %) were applied three times for miosis 20 min prior to PI surgery. Firstly, above the 12 o’clock position, a conjunctival incision was made 2 mm to the keratoconjunctival limus to dissect the lower conjunctival edge, forming a conjunctival flap with its bottom located at the conjunctival limus. Secondly, a 3-mm corneal incision was made at the corneal limbus beneath the conjunctival flap. Subsequently, iris forceps were inserted through the corneal incision to clamp and partially pull the surrounding iris outside the cornea; the iris was partially resected and the incision was about 1–2 mm. Finally, after repositioning the iris, the iris pigment layer was checked and the penetrating resection was performed. The conjunctival incision was closed with 10-0 suture.

Two weeks after undergoing the two aforementioned PI approaches, a 3.2-mm main corneal incision was made at the temporal side of the transparent corneal limbus for ICL implantation. Two auxiliary corneal incisions were made at the 12:00 and 6:00 positions. A routine implantation of the ICL lens was performed, followed by carbachol injection for miosis.

For intraoperative PI during ICL implantation, a 3.2-mm corneal incision was made at the 12:00 position during ICL implantation surgery. Two auxiliary corneal incisions were made at the 3:00 and 9:00 positions. The main corneal incision was shorter than a conventional corneal incision because the PI needed to be performed simultaneously. The ICL was implanted through the main corneal incision and then rotated to a horizontal orientation. After miosis with carbachol, the PI was performed at the 12:00 position. The diameter of each hole was approximately 1.0–2.0 mm. The corneal incision was sutured once or not at all based on the different conditions.

### Outcomes

The primary outcome was the time to eyesight recovery. The secondary outcome was the number of complications (pigment dispersion, iris bleeding, elevated IOP, etc.).

### Postoperative care and follow-up

TobraDex was given four times/day for 3 days after YAG PI and surgical PI. All patients received applications of TobraDex four times/day for 3 days after ICL implantation. The dosage of TobraDex was reduced once every 4 days and stopped 16 days after ICL implantation. Levofloxacin eye drops were applied four times/day for 1 week after ICL implantation.

Follow-up was completed 1 day, 1 week, and 2 weeks after YAG PI and surgical PI, and 1 day, 1 week, 1 month, and 3 months after ICL implantation. Follow-up examinations included assessments of IOP, BCVA, postoperative recovery of uncorrected visual acuity (UCVA) (logarithm of the minimum angle of resolution [LogMAR]), and patient’s acceptance and satisfaction.

### Complications

Complications were compared among the three iridectomy approaches and included iris bleeding, iris prolapses, pigment dispersion, visual disturbances, and elevated IOP (measured using noncontact tonometers). Pigment dispersion was qualitatively checked during slit-lamp examination. Normal dispersion appeared as scattered pigment deposition on the crystalline lens surface after preoperative PI or on the ICL surface after ICL implantation. The visual disturbance questionnaire was administered to the participants to inquire whether they had experienced any of the following either before or after the iridotomy or after ICL implantation: halo, diplopia, crescent, ghost image, glare, spots, shadows, blurring, or other visual disturbances. Patients with postoperative IOP >21 mmHg or an increase >5 mmHg over the preoperative IOP were considered to have elevated IOP.

### Statistical analysis

SPSS 13.0 statistical software (SPSS Inc., Chicago, IL, USA) was used for data analysis. Categorical data are expressed as frequency and were compared using the Chi-square test or the Fisher exact test, as appropriate. Normally distributed continuous variables are presented as mean ± standard deviation and were compared one-way analyses of variance (ANOVA). Paired *t* tests were used to compare pre- and postoperative visual acuity. *P* < 0.05 was considered statistically significant.

## Results

### Baseline characteristics of the patients

Fifty-eight patients were included in the study, and 12 patients were lost to follow-up (four patients in the YAG PI before ICL implantation group; three patients in the PI before ICL implantation group; five patients in the PI during ICL implantation). Finally, 46 completed the study, including 15 patients that underwent YAG PI before ICL implantation group (30 eyes), 17 patients (34 eyes) that underwent PI before ICL implantation group, and 14 patients (28 eyes) that underwent PI during ICL implantation. The characteristics of the patients are presented in Table 1. The patients were aged 19–41 years (*P* = 0.65 among the three groups). Gender distribution was similar (*P* = 0.16 among the three groups). There was no significant difference in diopter of the patients’ eyes and IOP before therapy among the groups (*P* = 0.43 and *P* = 0.69, respectively, among the three groups).

**Table 1:** Demography and clinical characteristics of the patients with high myopia and dark brown irides according with the different peripheral iridectomy (PI) approaches

Variables	YAG PI before ICL implantation (*n* = 15)	PI before ICL implantation (*n* = 17)	PI during ICL implantation (*n* = 14)	*P*
Age (mean ± SD) (range), years	25.9 ± 7.4 (19–41)	26.5 ± 4.9 (19–36)	26.0 ± 5.8 (19–35)	0.65
Sex (M/F)	8/7	10/7	8/6	0.16
Diopter	−14.44 ± 3.54	−14.17 ± 2.88	−14.72 ± 3.35	0.43
IOP (mean ± SD), mmHg	12.5 ± 1.4	13.2 ± 1.8	13.4 ± 2.1	0.69

YAG PI before implantable contact lens (ICL) implantation group: Neodymium:yttrium-aluminum-garnet (Nd:YAG) laser peripheral iridectomy (YAG PI) was performed 2 weeks prior to the ICL implantation. PI before ICL implantation group: PI was performed 2 weeks prior to the ICL implantation. PI during ICL implantation group: PI was performed during ICL implantation

*ICL* implantable contact lenses, *SD* standard deviation, *M* male, *F* female

### Visual acuity recovery

There was no significant difference of the preoperative BCVA among the three groups (all *P* > 0.05) (Table 2). On postoperative day 1 after YAG PI, two of the 30 eyes (6.7 %) in the YAG PI group had a decreased BCVA caused by incomplete absorption of blood in the anterior chamber. On postoperative 1 day after ICL implantation, the UCVA in the YAG PI group and the preoperative PI group achieved the same level compared to preoperative BCVA (*P* = 0.22 and *P* = 0.53, respectively). However, in the intraoperative PI group, the UCVA was significantly lower than the preoperative BCVA (*P* = 0.04) on the first day after ICL implantation and recovered to the preoperative level at 1 week (*P* = 0.67).

**Table 2:** Recovery of UCVA post-ICL Implantation for the different PI approaches

Group	Number of eyes	Pre-operative BCVA	Post-operative UCVA			
			1 day	1 week	1 month	3 months
YAG PI before ICL implantation	30	0.55 ± 0.20	0.48 ± 0.10	0.63 ± 0.13	0.75 ± 0.20	0.80 ± 0.21
*P* (vs. preoperative BCVA)			0.22	0.10	0.006	<0.001
PI before ICL implantation	34	0.53 ± 0.10	0.51 ± 0.12	0.67 ± 0.19	0.76 ± 0.24	0.78 ± 0.23
*P* (vs. preoperative BCVA)			0.53	0.004	<0.001	<0.001
PI during ICL implantation	28	0.56 ± 0.15	0.46 ± 0.16	0.54 ± 0.13	0.75 ± 0.20	0.80 ± 0.18
*P* (vs. preoperative BCVA)			0.04	0.67	<0.001	<0.001
*P*_*1*_		0.73	0.41	0.76	0.91	0.81
*P*_*2*_		0.84	0.63	0.02	0.97	1.00
*P*_*3*_		0.46	0.24	0.01	0.87	0.78

*Note* Data are shown as mean ± SD

Pre-operative BCVA and postoperative UCVA was present as logarithm of the minimum angle of resolution [LogMAR]

*BCVA* best-corrected visual acuity, *UCVA* uncorrected visual acuity

*P*_*1*_: YAG PI before ICL implantation vs. PI before ICL implantation

*P*_*2*_ *:* YAG PI before ICL implantation vs. PI during ICL implantation

*P*_*3*_: PI before ICL implantation vs. PI during ICL implantation

Compared with the preoperative BCVA, the UCVA at 1 week was markedly restored in the preoperative PI group (*P* = 0.004). However, there was no significant difference compared with the YAG PI group (*P* = 0.76). The improvements in the UCVA were better in the preoperative PI and YAG PI groups compared with the intraoperative PI group 1 week after ICL implantation (both *P* < 0.05). The UCVA in the three groups of patients had all recovered well at 1 and 3 months after ICL implantation and were significantly better than the preoperative BCVA (all *P* < 0.01) (Table 2).

### Complications

Surgical complications are presented in Table 3. In the YAG PI group, nine of the 30 eyes (30.0 %) had iris bleeding during laser application, and the laser treatment had to be stopped for two eyes (one eye in two patients). TobraDex was administered, and both eyes were bandaged. Laser treatment was performed the next day after the blood was partially absorbed. Among the 34 eyes in the preoperative surgical PI group, two eyes (5.9 %) had a small amount of bleeding during surgical PI, which was significantly less than in the YAG PI group (*P* = 0.01) (Table 3). Follow-up exams on postoperative day 1 showed that bleeding was completely absorbed. None of the patients experienced iris bleeding during ICL implantation.

**Table 3:** Comparison of iridectomy complications, ICL implantation complications, and duration of ICL implantation operations in different PI approach

Variables	YAG PI before ICL implantation	PI before ICL implantation	PI during ICL implantation	*P* ^a^
Number of eyes	30	34	28	
Iridectomy Complications, n (%)				
Iris bleeding	9 (30.0)	2 (5.9)	-	0.01
Pigment dispersion	14 (46.7)	5 (14.7)	-	0.01
Elevated IOP	6 (20.0)	2 (5.9)	-	0.08
Decreased BCVA	2 (6.7)	0 (0.0)		0.22
ICL Implantation Complications, n (%)				
Iris prolapse	0 (0.0)	0 (0.0)	2 (7.1)	1.00
Pigment dispersion	2 (6.7)	3 (8.8)	8 (28.6)	0.34
Visual disturbances	2 (6.7)	1 (2.9)	0 (0.0)	0.35
Iris bleeding	0 (0.0)	0 (0.0)	0 (0.0)	1.00
Elevated IOP	2 (6.7)	1 (2.9)	4 (14.3)	0.35
Duration of ICL implantation operation, min	27.2 ± 5.7	29.8 ± 5.6	45.3 ± 7.0	0.06

*Note*: Data are shown as mean ± SD or proportion, as appropriate

^a^PI before ICL implantation vs. YAG PI before ICL implantation

-: Not assessed. IOP: intraocular pressure

The IOP and BCVA were assessed on postoperative 1 day

Elevated IOP: postoperative IOP >21 mmHg or IOP increased by >5 mmHg over the preoperative IOP

Among the 28 eyes in the intraoperative PI during ICL implantation group, iris prolapse occurred in two eyes (7.1 %). Both were successfully repositioned.

Fourteen eyes (46.7 %) in the YAG PI group had a varying degree of pigment dispersion that almost completely disappeared by the time of ICL surgery 2 weeks later. Among the 34 eyes in the preoperative PI group, five eyes (14.7 %) had a small amount of pigment dispersion, which was less than in the YAG PI group and was absorbed more quickly (*P* = 0.01) (Table 3). The dispersed pigment had almost completely disappeared 1 week after surgery. Only two eyes in the YAG PI group and three eyes in the preoperative PI group had a small amount of iris pigment dispersion during ICL implantation, which was completely absorbed in the first week after surgery.

Only two individuals in this study reported visual disturbances. One saw shadows in one eye immediately after preoperative surgical PI, but it had improved at the following follow-up examination. This patient had an exposed iridotomy. The other patient had received YAG PI and complained of glare in both eyes after ICL implantation, but this improved over time.

None of the patients experienced pupillary block. In the YAG PI group, six of the 30 eyes (20.0 %) had an elevated IOP on postoperative 1 day (IOP >21 mmHg or an increase >5 mmHg compared with baseline); the highest IOP value was 25 mmHg. No medication was given, and the IOP returned to a normal level 1 week after surgery. Two eyes from the YAG PI group had increased IOP 1 day after ICL implantation (22 and 23 mmHg, respectively), and recovered on postoperative week 1 without any medication. However, the other two eyes had increased IOP during the first postoperative week of examination (21 and 23 mmHg, respectively). A steroid-induced elevation of IOP was considered, and IOP returned to normal after cessation of steroid hormone. In the preoperative surgical PI group, elevated IOP was observed in two eyes after the preoperative PI and was noted in only one eye on the first day after ICL implantation (23 mmHg) (Table 3). The IOP returned to normal without special treatment. Four eyes (14.3 %) in the intraoperative PI during ICL implantation group had an elevated IOP on the first day after ICL implantation (19, 21, 22, and 24 mmHg, respectively). The IOP returned to normal 1 week after surgery without special treatment (Table 3).

### Operation duration

The duration of the ICL implantation operation in the YAG PI group was 27.2 ± 5.7 min (20–36 min) compared to 29.8 ± 5.6 min (21–39 min) in the preoperative PI group (*P* > 0.05). The operation duration of intraoperative PI during ICL was 45.3 ± 7.0 min (35–55 min) (Table 3).

## Discussion

These results strongly suggest that surgical PI performed 2 weeks prior to ICL implantation facilitates better postoperative recovery of visual acuity. The YAG PI method has been recommended by the STAAR Surgical Co. as one of the main approaches for PI, but the majority of previous surgeries were performed on European patients, whose irides are typically blue or gray and thus contain less pigment than those of Asian patients. In addition, the irides of Europeans are generally thinner compared with those of Asians, making them easier to access via laser. In Asian patients, it is difficult to simultaneously make two large, unblocked holes due to severe pigment dispersion, which is considered to be a strong risk factor for increased IOP [13]. Brandt et al. [14] and Sanchez-Galeana et al. [15] described cases with pigmentary glaucoma secondary to implantation of a phakic posterior chamber intraocular lens. Chun et al. [11] indicated that the pigment in all quadrants increased because of laser iridotomy when performed on dark-brown irides. When the PI is performed, mechanical injury caused by scissors is less than that caused by the YAG laser. In this study, the incidence of pigment dispersion in the preoperative PI group was significantly less compared with that in the YAG PI group.

Rosen et al. [2] reported that patients with brown irides were more likely to develop a pupillary block, but the reason was not defined in the article. In the study by Chun et al. [11], peripheral iridotomies were gradually occluded or narrowed by ≥50 % in ten eyes (12.3 %), with regeneration of dark-brown iris pigment epithelium around the laser iridotomy opening. The authors hypothesized that this may have been due to sterile chronic inflammation from a compromised anterior blood-ocular barrier. Therefore, the iridotomy size in dark-brown irides for ICL implantation should be slightly larger than that used in a conventional laser iridotomy. It requires a high level of skill to use a laser to make holes in a dark-brown iris. Improper operation of a YAG laser can cause iatrogenic trauma to the crystalline lens and zonules [12, 16]. Pesando et al. [12] also stopped using laser treatment on patients with brown irides; rather, they used a traditional iridectomy and achieved good results. The incision for surgical PI is much larger than that of the YAG PI, making it difficult to develop a viscoelastic material-induced obstruction and iridectomy closure can be caused by iris edema, postoperative inflammation, exudative membrane blocking, and pigment granule block [7]. Therefore, surgical PI is theoretically more effective in preventing pupillary block. In this study, all three PI approaches successfully prevented pupillary block.

Selection of the iridotomy site is particularly important for preventing visual disturbances after PI [10]. The traditional PI sites recommended by the STAAR Surgical Company are located approximately 90° apart at 10:30 and 1:30 positions. Although care was taken to locate the PI sites under the eyelids, the incidence of the visual disturbances was 7.0–9.9 % in previous reports [11, 17]. Spaeth et al. [17] reported that most of the iridotomies placed outside the 11:00 to 1:00 range were fully or partially exposed, which put the patients at a higher risk for visual disturbances than if the iridotomy had been fully covered by the eyelids. Theoretically, a corneal incision made at the 12:00 position would have good upper eyelid coverage, and this prevents glare or double vision. However, locating the iridotomy at the 12:00 position does not assure complete coverage of the iridotomy. In eyes with lid retraction, it is important to consider carefully the likelihood of visual disturbances. We did not observe a significant difference between the YAG PI and surgical PI approaches.

It is recommended in the traditional YAG PI that two peripheral iridotomies should be located superiorly approximately 90° apart to decrease the likelihood of occlusion by the ICL haptics [1, 18]. However, variations on this principle have also been described. In his later cases, Gonvers et al. [10] performed only one iridotomy on the 12:00 meridian. For surgical PI, the corneal incision is also made at the 12:00 position. Because the current ICL and T-ICL lenses are designed for a horizontal orientation, the ICL lens cannot move on the lens surface after it is in place. Therefore, it is theoretically impossible for the lens edge to block the PI incision at the 12:00 position.

Iris bleeding can necessitate a second laser surgery or lead to vision loss, which will increase mental and financial burden on the patients, especially those with inconvenient transportation options or high transportation costs. Surgical PI is a brief and simple procedure, which would achieve good predictability of the outcome and postoperative recovery. The patients had no obvious discomfort on the following day, and they had no changes in their vision during this study. Therefore, all of the patients in the preoperative surgical PI group in the present study were more satisfied than those in the other groups.

A single surgery to complete peripheral iridectomy and ICL implantation can reduce the psychological and economic burden to patients. However, due to the extended surgery duration and complex operating procedure, the inflammatory responses to the surgery are more serious. Studies from a U.S. clinical research team using ICL to treat high myopia [19] and results reported Jiménez-Alfaro et al. [20] described that the ICL postoperative inflammatory reaction was mainly caused by the surgery itself. Therefore, a shorter surgery duration and simpler operating procedure may result in less injury, a reduced postoperative reaction, and better vision recovery. In this study, it was more likely for the patients to develop iris prolapse in the intraoperative PI during ICL implantation group because of the shorter main corneal incision, although there was no statistical significance compared with the other two PI methods (*P* > 0.05). The surgeon must be highly skilled to reduce the operation duration and decrease the rate of complications.

There are some limitations to this study. It was performed at a single institution, so the numbers of patients in the three groups were relatively small. In addition, all of our patients were healthy, so it is unclear if similar effects would be achieved in patients with comorbidities. In addition, for the third operation approach, the ICL had to be rotated during the operation and this could increase the risk of damage; however, plenty of viscoelastic agent was injected to protect the crystal during operation and there was no case of complication induced by rotation. Thus, rotation was practical if the operation was carefully conducted. Finally, during the design and process of the present study, the newest central hole type ICL V4c, which is designed to eliminate the need for PI, was not on the market yet. Nevertheless, further large-scale studies are necessary to confirm our findings.

## Conclusions

The results of the present study suggest that it might be preferable that patients with dark-brown irides undergo surgical PI 2 weeks prior to ICL implantation. Different PI methods should be selected according to each patient characteristics (e.g., iris color and thickness, and corneal pannus extent), patient transportation and economic situations, hospital situations, and surgeon proficiency to minimize complications, reduce patient stress, and financial burden, and to achieve the greatest degree of patient satisfaction.

## Ethics

The study was approved by the ethical committee of the ChengDu Military General Hospital (Sichuan, China). A written informed consent was obtained from participant. The study was carried out in accordance to the principle of the Declaration of Helsinki.

## Consent to publish

Not applicable.

## Availability of data and materials

The dataset supporting the conclusions of this article is included within the article.

## Abbreviations

ANOVA: one-way analyses of variance
BVCA: best-corrected visual acuity
ICL: implantable contact lenses
IOP: intraocular pressure
Nd:YAG: neodymium: yttrium-aluminum-garnet
PI: peripheral iridectomy
UCVA: uncorrected visual acuity
